# Gallic Acid Improves Diabetic Steatosis by Downregulating MicroRNA-34a-5p through Targeting NFE2L2 Expression in High-Fat Diet-Fed db/db Mice

**DOI:** 10.3390/antiox11010092

**Published:** 2021-12-30

**Authors:** Ang-Tse Lee, Mon-Yuan Yang, Yi-Ju Lee, Tzu-Wei Yang, Chi-Chih Wang, Chau-Jong Wang

**Affiliations:** 1 Institute of Medicine, Chung Shan Medical University, Taichung 402, Taiwan; lavenoh@gmail.com (A.-T.L.); koiyung@gmail.com (M.-Y.Y.); 2 Wang Chin-Yao Clinic, Taichung 408, Taiwan; 3 Department of Pathology, Chung Shan Medical University Hospital, Taichung 402, Taiwan; jasmine.lyl@gmail.com; 4 Department of Pathology, Chung Shan Medical University, Taichung 402, Taiwan; 5 Division of Gastroenterology and Hepatology, Department of Internal Medicine, Chung Shan Medical University Hospital, Taichung 402, Taiwan; joviyoung@gmail.com; 6 School of Medical, Chung Shan Medical University, Taichung 402, Taiwan; 7 Department of Health Industry Technology Management, Chung Shan Medical University, Taichung 402, Taiwan; 8 Department of Medical Research, Chung Shan Medical University Hospital, Taichung 402, Taiwan

**Keywords:** microRNA, lipogenesis, diabetic steatosis, gallic acid, NFE2L2

## Abstract

Type 2 diabetes mellitus (T2DM) has become epidemic worldwide and is strongly associated with nonalcoholic fatty liver disease (NAFLD). The molecular mechanisms of microRNAs in NAFLD and T2DM development and the corresponding therapies remain unclear. We performed microRNA microarray validation to determine whether hepatic miR-34a-5p was significantly upregulated in db/db mice fed with a high-fat diet (HFD), a mouse model of T2DM with steatohepatitis. The potential role of miR-34a-5p and gallic acid (GA) in regulating hepatic lipid metabolism and diabetic steatosis was explored. GA improved the activities of antioxidant enzymes and suppressed lipid accumulation in the HFD-induced steatotic liver of db/db mice. In vitro, the silencing of miR-34a-5p in hepatocyte HepG2 cells ameliorated high glucose + oleic acid/palmitic acid mixture–induced accumulation of cellular triglycerides. We identified nuclear factor erythroid-derived 2-like 2 (NFE2L2) as a direct target of miR-34a-5p. Reduction in intracellular triglyceride and the expression levels of sterol regulatory element-binding protein 1 and fatty acid synthase by GA were mediated by the inhibition of miR-34a-5p expression in HepG2 cells. The findings suggest that GA improves hepatic lipogenesis by downregulating miR-34a-5p by suppressing NFE2L2 expression, indicating the potential therapeutic role of GA or an NFE2L2-activating agent in diabetic fatty liver disease.

## 1. Introduction

Type 2 diabetes mellitus (T2DM) is associated with various complications such as retinopathy, diabetic kidney disease, diabetic neuropathy, and nonalcoholic fatty liver disease (NAFLD), which is characterized by the excessive accumulation of triglycerides in the liver and encompasses both simple steatosis and nonalcoholic steatohepatitis (NASH). Dysregulation of lipogenesis tends to increase lipid accumulation in the liver, leading to insulin resistance and T2DM. Therefore, NAFLD is correlated with T2DM, obesity, insulin resistance, oxidative stress, and inflammation, and NAFLD was identified as an early predictor of T2DM in a prospective cohort study [1,2].

MicroRNAs (miRNAs or miRs) are endogenous noncoding single-stranded RNAs (approximately 22 nucleotides in length) that downregulate gene expression through posttranscriptional regulation [3]. Different miRNA clusters have been identified as crucial regulators of hypertension, insulin resistance, and atherogenic dyslipidemia in metabolic syndrome [4]. Several miRNAs alter hepatic lipid metabolism and regulation during the development and progression of steatosis through the modulation of molecular and epigenetic mechanisms by targeting specific sites [5,6]. Liver miR-291b-3p caused lipid accumulation by inhibiting adenosine monophosphate-activated protein kinase (AMPK)-α1 expression in mice [7]. Given the pivotal role of miR-122 in regulating lipid metabolism, inhibition of miR-122 in high-fat diet (HFD)-induced obese mice reduced the plasma cholesterol level and ameliorated liver steatosis [8]. Moreover, miR-34a is a crucial regulator of insulin secretion, glucose metabolism, and hepatic lipid metabolism [5,6,9]. Hepatic miR-34a overexpression was observed in NAFLD, and miR-34a upregulation caused steatosis by downregulating peroxisome proliferator-activated receptor alpha (PPARα) and sirtuin 1 (SIRT1) [10].

Polyphenols can regulate miRNAs during inflammation, apoptosis, carcinogenesis, lipid metabolism, and cell migration [11]. Among various types of naturally occurring polyphenols, gallic acid (GA) can modulate various human diseases through its antioxidative, anti-inflammatory, and antiproliferative activities [12,13,14]. In addition, nuclear factor erythroid-derived 2-like 2 (NFE2L2), also known as nuclear factor erythroid 2-related factor 2 (NRF2), is a member of the Cap’n’Collar family of basic leucine zipper transcription factors. NFE2L2 is a key player in cellular resistance to oxidants, and it controls many antioxidative responses [15]. Oxidative stress and inflammation play crucial roles in NAFLD regulation [16]; however, the molecular mechanisms of the role of miRNAs in diabetic steatosis development and their therapies have not yet been investigated. This study examined the potential role of miR-34a-5p and GA in regulating hepatic lipid metabolism and diabetic steatosis. We observed that miR-34a-5p directly targeted the transcription factor NFE2L2, which regulates hepatic lipogenesis, and that GA exerted a therapeutic effect on HFD-fed diabetic mice.

## 2. Materials and Methods

### 2.1. Chemicals and Reagents

GA; ethylenediaminetetraacetic acid; 3-(4,5-dimethylthiazol-2-yl)-2,5 diphenyl- tetrazolium bromide; Oil Red O; paraformaldehyde; propidium iodide; ribonuclease A; sodium bicarbonate; peroxidase-conjugated antibodies against goat IgG, mouse IgG, and rabbit IgG; and β-actin (1:10,000, A5441, as the internal control) were purchased from Sigma (St. Louis, MO, USA). AMPK (1:500, sc-74461), carnitine palmitoyltransferase I (CPT-I, 1:500, sc-20669), sterol regulatory element-binding protein 1 (SREBP1, 1:500, sc-13551), SREBP2 (1:500, sc-5603), and a phosphorylated signal transducer were purchased from Santa Cruz Biotechonology (Santa Cruz, CA, USA). pAMPK (1:1000, #2535) and fatty acid synthase (FASN, 1:1000, #3180) were purchased from Cell Signaling Techonology (Beverly, MA, USA). 3-hydroxy-3-methyl- glutaryl-coenzyme A (HMG-CoA, 1:500, E-AB-11788) reductase and PPARα (1:5000, E-AB-32646) were procured from Elabscience (Texas, TX, USA). 1-acylglycerol-3-phosphate-*O*-acyltransferase 1 (AGPAT1, 1:1000, ab67018) was purchased from Abcam (Cambridge, UK).

### 2.2. Animal Maintenance and Treatment

The BKS.Cg-*Dock7^m^* +/+ *Lepr^db^*/JNarl (db/db) mice aged 4 to 6 weeks and weighing approximately 20 g were purchased from the National Laboratory Animal Center (Taipei, Taiwan), and they were originally obtained from The Jackson Laboratory. The mice were maintained in the animal housing room under a 12-h light/12-h dark cycle in a temperature (22 °C ± 2 °C)-controlled environment. All animal protocols were approved by the Institutional Animal Care and Use Committee of Chung Shan Medical University (IACUC, CSMU, approval no. 2025). db/db mice, an animal model of T2DM, have hyperglycemia, hyperinsulinemia, hypertriglyceridemia, and insulin resistance [17,18]. db/m mice are age-matched heterozygotes without diabetes. These mice were divided into four groups: (1) the db/m group (n = 3) as the control group (they were fed with Standard Laboratory Rodent Diet 5010 containing 24.6% protein, 5.0% fat, 4.2% crude fiber, and 6.1% ash for 12 weeks according to the manufacturer’s instructions); (2) the db group (n = 3; db/db mice were fed with a standard diet as group 1 for 12 weeks); (3) the HFD group (n = 3; db/db mice were fed with HFD [15% lard oil and 0.5% cholesterol supplemented in diet] for 12 weeks); and (4) the GA group (n = 3; db/db mice were fed with HFD and received the intragastric administration of GA at 100 mg/kg until week 12). At week 12, the mice from each group were anaesthetized by Zoteil 50 (12.5 mg/kg), infused carbon dioxide for 5 min, and then sacrificed. The blood samples were collected through cardiac puncture for biochemical analysis. The livers were harvested for the measurement of weight and the pathological determination of liver steatosis.

### 2.3. Determination of Plasma Biomarkers for Liver Steatosis and Relevant Biochemical Analysis

Plasma levels of aspartate aminotransferase (AST; AS521, Randox Laboratories, Antrim, UK), alanine aminotransferase (ALT; AL520, Randox Laboratories), total cholesterol (CH201, Randox Laboratories), and triglycerides (TR213, Randox Laboratories) were determined through enzymatic colorimetric methods using commercial kits (Randox Laboratories). In addition, levels of insulin (Mercodia AB, Uppsala, Sweden) and glycosylated hemoglobin (HbA1c; Fortress Diagnostics, UK) were determined.

### 2.4. Measurement of Lipid Peroxidation and Antioxidant Enzymes 

Lipid peroxidation was determined by measuring the amount of malondialdehyde that was reacted with thiobarbituric acid to form thiobarbituric acid reactive substances (TBARS) [19]. The absorbance of the supernatant was measured at 532 nm. TBARS levels are presented as mmol/mg protein. The amount of glutathione (GSH) utilized in the assay mixture was measured before and after enzymatic activity (μg/mg protein). GSH peroxidase (GSH Px) activity (nmol NADPH/min/mg protein) was spectrophotometrically determined using the method reported by Lawrence and Burk [20]. GSH reductase (GSH Rd) activity (nmol NADPH/min/mg protein) was measured using phosphate-buffered saline (containing 1.1 mM MgCl_2_·6H_2_O, 5.0 mM glutathione disulfide, and 0.1 mM NADPH) at 340 nm. Superoxide dismutase (SOD) activity (U/mg protein) was calculated based on the inhibition of 0.2 mM pyrogallol autooxidation (U = 50% inhibition of activity). Catalase activity (U/mg protein) was measured using 3 mM H_2_O_2_ at 240 nm.

### 2.5. Western Blotting

Liver cells were lysed with RIPA lysis buffer after treatment with reagents for 24 h. The lysates were centrifuged, and the supernatant was collected. Equal amounts of protein samples (50 μg) were subjected to sodium dodecyl sulfate–polyacrylamide gel electrophoresis and subsequently electrotransferred onto nitrocellulose membranes (Millipore, Bedford, MA, USA). The membranes were incubated with a blocking solution (5% nonfat milk powder with 0.1% Tween 20 in Tris-buffered saline (TBS)) and subsequently incubated with the indicated primary antibody at 4 °C overnight. Thereafter, the membranes were washed three times with 0.1% Tween 20 in TBS and incubated with a horseradish peroxidase–conjugated second antibody (GE Healthcare, Little Chalfont, Buckinghamshire, UK). Finally, protein bands were detected using enhanced chemiluminescence (ECL) and an exposed ECL hyperfilm in Fujifilm LAS-4000 (Tokyo, Japan). Protein quantitation was performed through densitometry by using the Fujifilm-Multi Gauge V2.2 software (Tokyo, Japan).

### 2.6. Histological Analysis of Tissues

The liver specimens were collected and fixed in 10% buffered neutral formalin for Oil Red O or hematoxylin and eosin (H&E) staining. For H&E staining, the slides were first incubated with hematoxylin for 30 s. After washing with water, the slides were stained with eosin for 2–5 min and dehydrated in an alcohol gradient. For Oil Red O staining, the tissues were incubated with prewarmed Oil Red O for 30 min at 37 °C and washed with 60% 1, 2-propanediol. Subsequently, histopathological changes in lipid droplets stained with H&E and Oil Red O were visualized using a light microscope.

### 2.7. Cell Treatment

HepG2 cells were treated with a mixture of 300 μM oleic acid and palmitic acid (O/P) (2:1, M/M) to induce hepatic steatosis and inflammation.

We purchased miRNA mimics, miRNA inhibitors, and the negative control from GeneDireX (Las Vegas, NV, USA). Transfection was performed using T-Pro nonliposomal transfection reagent II (T-Pro NTR II, T-Pro Biotechnology, Taipei, Taiwan).

### 2.8. miRNA Isolation and Real-Time Polymerase Chain Reaction

Total RNA was extracted from the liver tissues. RNA was transcribed to cDNA, and stem-loop reverse transcription-polymerase chain reaction (RT-PCR) was conducted. Real-time PCR was performed using the LightCycler 480 SYBR Green I Master mix and Light Cycler 480 real-time PCR machine (Roche Applied Science). The relative expression level of an miRNA was normalized to that of an internal control. The relative expression level of an miRNA was normalized to that of an internal invariant control, RNU6B (6B). Each reaction was performed in triplicate, and analysis was performed using the 2^−ΔCt^ method.

Nucleotide primers (5′-3′) used for reverse transcription were GTTGGCTCTGGTGCAGGGTCCGAGGTATTCGCACCAGAGCCAACACAACC for miR-34a-5p and GTGCAGGGTCCGAGGTATTCGCACCAGAGCCAACAAAAATAT for 6B.

The primers (5′-3′) used for real-time PCR were as follows: miR-34a-5p forward, CGATTGGCAGTGTCTTAGCT; 6B forward, TTCCTCCGCAAGGATGACACGC; and universal reverse primer, GTGCAGGGTCCGAGGT.

### 2.9. Luciferase Reporter Assay

Luciferase activity was determined using miRNA 3′-UTR target clones from GeneCopoeia miTarget™ designed for the identification of miRNAs and the functional validation of predicted targets. The 3′-UTR sequences were obtained from a gene sequence database, and a secreted *Gaussia* luciferase (GLuc) reporter gene was inserted. Chimeric mRNA consisting of GLuc and a 3′-UTR target sequence was transcribed [21]. Subsequently, the mRNA–miRNA target interaction was mediated through a live cell assay for GLuc by using only 10 μL of the cell culture medium. In addition to using GLuc as the miRNA 3′-UTR target reporter, we cloned secreted alkaline phosphatase (SEAP), which served as the internal control. Thus, the dual-reporter system—GLuc and SEAP—was established and the 3′-UTR sequence of *NFE2L2* (gene accession no. NM_001145413.2) was predicted to interact with miR-34a-5p.

### 2.10. Statistical Analysis

The results are presented as the mean ± standard deviation (SD). Analysis of variance (ANOVA) was performed. Significant differences (*p* < 0.05) among the groups were determined using Duncan’s multiple range test (Sigma-Plot 12.0, Jandel Scientific, San Rafael, CA, USA) [22].

## 3. Results

### 3.1. GA Improved Liver Function, Hyperlipidemia, Hyperinsulinemia, and Hepatic Steatosis in Diabetic Mice

**Figure 1:**
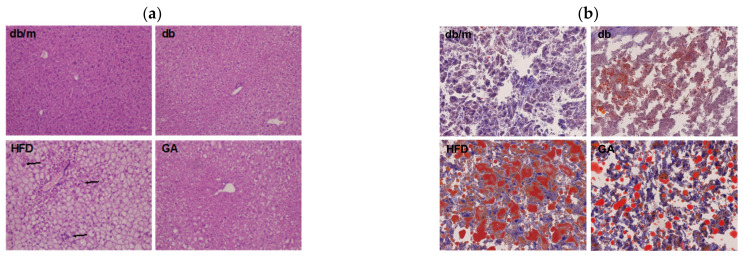
HFD induced hepatic steatosis, and GA improved lipid accumulation in diabetic mice. 6-week-old male db/db mice fed with HFD and 100 mg/kg GA for 12 weeks. (**a**) H&E (100×) and (**b**) Oil Red O staining (200×) of liver frozen sections. The accumulation of polymorphonuclear leukocytes is indicated with an arrow. db/m, age-matched heterozygous mice fed with standard diet group; db, db/db mice fed with standard diet group; HFD, db/db mice fed with HFD group; GA, db/db mice fed with HFD and GA group.

To examine glucose and lipid homeostasis, we analyzed plasma biochemical parameters and hepatic lipid levels in mice. The HFD group had higher plasma levels of AST, ALT, insulin, cholesterol, and triglycerides than did the db group (Table 1). These results indicated that HFD led to impaired liver function, hyperlipidemia, and hyperinsulinemia. The GA group exhibited significant improvement in these metabolic parameters compared with the HFD group mice. Although no significant changes in HbA1c were noted after feeding the mice with a HFD or GA, the HFD-fed diabetic mice that received GA displayed a recovering trend of hyperinsulinemia. In addition, HFD increased the weight of the liver and promoted the accumulation of numerous lipid droplets and polymorphonuclear leukocytes in the liver, as observed through H&E and Oil Red O staining (Figure 1). These results suggest that HFD damaged the liver parenchyma with hypertrophy of adipocytes, leading to hepatic steatosis accompanied by worsening of liver function. The administration of GA for 12 weeks contributed to the improvement of liver function and resulted in a significant reduction in excessive fat deposition in hepatic intracellular vacuoles, thus indicating the hepatoprotective effects of GA on HFD-induced steatosis and inflammation. Taken together, the results showed that GA administration improved lipid homeostasis by protecting against hyperlipidemia, hepatic steatosis, and hyperinsulinemia in HFD-induced NAFLD mice.

### 3.2. GA Ameliorated Hepatic Lipid Peroxidation and Enhanced the Activities of Hepatic Antioxidant Enzymes in Diabetic Mice

**Figure 2:**
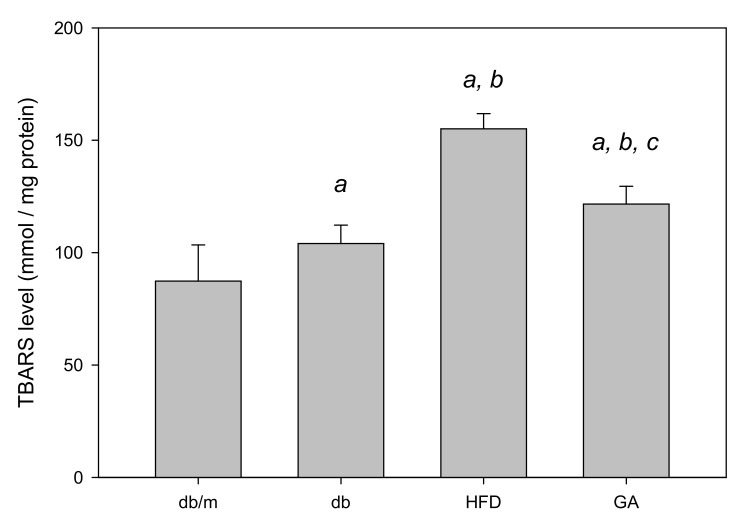
HFD induced hepatic lipid peroxidation and GA ameliorated it in diabetic mice. 6-week-old male db/db mice fed with HFD and 100 mg/kg GA for 12 weeks. Thiobarbituric acid reactive substances (TBARS) levels of the liver were analyzed. db/m, age-matched heterozygous mice fed with standard diet group; db, db/db mice fed with standard diet group; HFD, db/db mice fed with HFD group; GA, db/db mice fed with HFD and GA group. a, *p* < 0.05 compared with the db/m control group. b, *p* < 0.05 compared with the db group. c, *p* < 0.05 compared with the HFD group.

**Figure 3:**
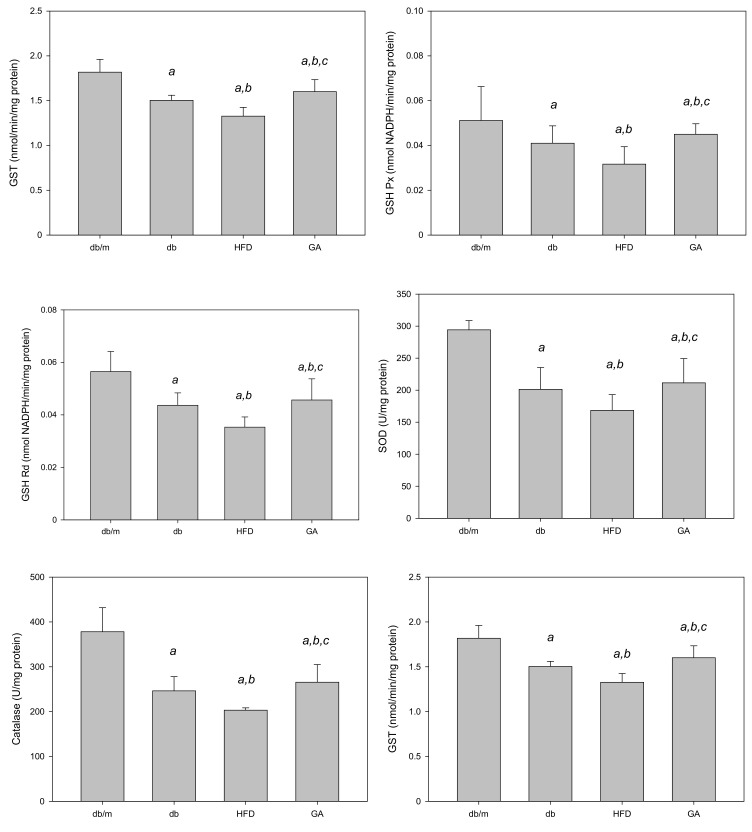
HFD reduced and GA enhanced the activities of hepatic antioxidant enzymes in diabetic mice. The 6-week-old male db/db mice were fed with HFD and 100 mg/kg GA for 12 weeks. Activity levels of GSH, GSH Px, GSH Rd, SOD, catalase and GST in the liver were analyzed. db/m, age-matched heterozygous mice fed with standard diet; db, db/db mice fed with standard diet; HFD, db/db mice fed with HFD; GA, db/db mice fed with HFD and GA. a, *p* < 0.05 compared with the db/m control group. b, *p* < 0.05 compared with the db group. c, *p* < 0.05 compared with the HFD group.

The TBARS assay was performed to evaluate the extent of lipid peroxidation. The TBARS level in the liver was significantly increased in the HFD group compared with the db group; however, GA treatment reduced the TBARS level (Figure 2). The levels of antioxidant enzymes, namely GSH, GSH Px, GSH Rd, SOD, catalase, and glutathione S-transferase (GST), were significantly decreased in the HFD group compared with the db group. GA treatment increased the levels of hepatic antioxidant enzymes (Figure 3). These findings indicated that HFD exacerbated lipid peroxidation and inhibited antioxidant enzymatic activities in the liver. Moreover, GA treatment effectively ameliorated lipid peroxidation and could enhance antioxidative effects.

### 3.3. GA Inhibited the Expression of Hepatic Lipid Metabolism–Related Proteins in Diabetic Mice

**Figure 4:**
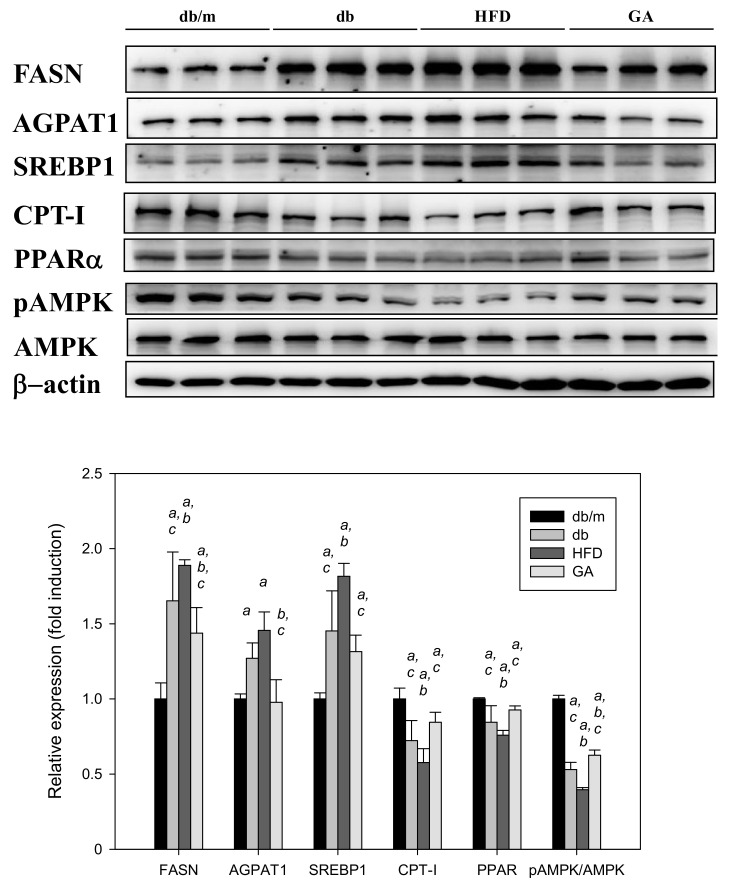
HFD induced and GA inhibited the expression of hepatic lipid metabolism–related proteins in diabetic mice. 6-week-old male db/db mice fed with HFD and 100 mg/kg GA for 12 weeks. After euthanization, liver tissue samples were obtained for determination with SREBP1 and FASN antibodies. db/m, age-matched heterozygous mice fed with standard diet; db, db/db mice fed with standard diet; HFD, db/db mice fed with HFD; GA, db/db mice fed with HFD and GA. a, *p* < 0.05 compared with the db/m control group. b, *p* < 0.05 compared with the db group. c, *p* < 0.05 compared with the HFD group.

The db/db mice fed with HFD exhibited significantly elevated levels of FASN and SREBP1 compared with those fed with the standard diet. The AGPAT1 level tended to increase in the HFD group. CPT-I and PPARα levels were significantly decreased in the HFD group compared with the db group. By contrast, GA treatment mainly reversed the effects of HFD on the lipid metabolism–related proteins in the liver (Figure 4). These results suggest that HFD promoted the synthesis of free fatty acids and inhibited fatty acid β-oxidation in the liver, whereas GA markedly altered lipogenic enzyme levels in the HFD-fed db/db mice.

**Figure 5:**
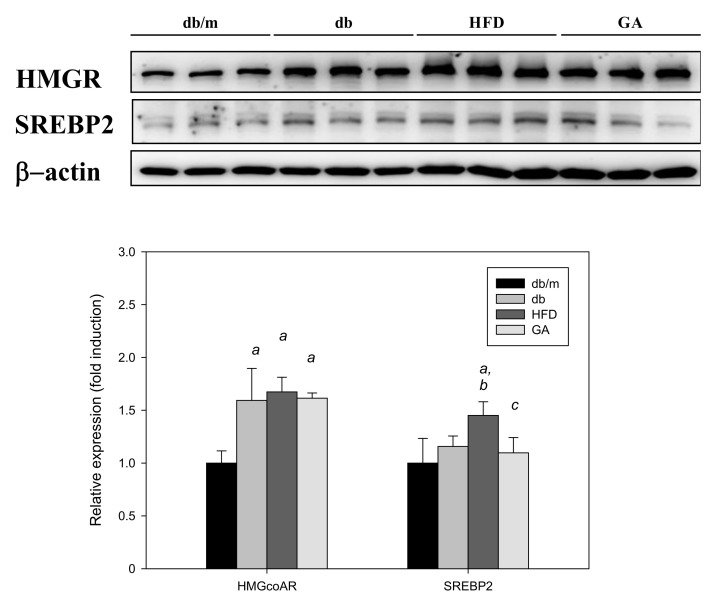
HFD induced the expression of cholesterol synthesis–related proteins and GA inhibited SREBP2 expression in diabetic mice. The 6-week-old male db/db mice were fed with HFD and 100 mg/kg GA for 12 weeks. After euthanization, liver tissue samples were obtained for determination with SREBP2 and HMG-CoA reductase antibodies. db/m, age-matched heterozygous mice fed with standard diet; db, db/db mice fed with standard diet; HFD, db/db mice fed with HFD; GA, db/db mice fed with HFD and GA. a, *p* < 0.05 compared with the db/m control group. b, *p* < 0.05 compared with the db group. c, *p* < 0.05 compared with the HFD group.

The SREBP2 level was significantly elevated in the HFD group compared with the db group, and GA treatment significantly inhibited the HFD-induced elevation of SREBP2 expression. However, the HMG-CoA reductase level did not significantly change after HFD or GA treatment. These results indicated that HFD or GA did not likely regulate hepatic lipid metabolism through the HMG-CoA reductase or SREBP2 pathway (Figure 5).

### 3.4. HFD Induced miRNA Expression in Diabetic Mice

**Figure 6:**
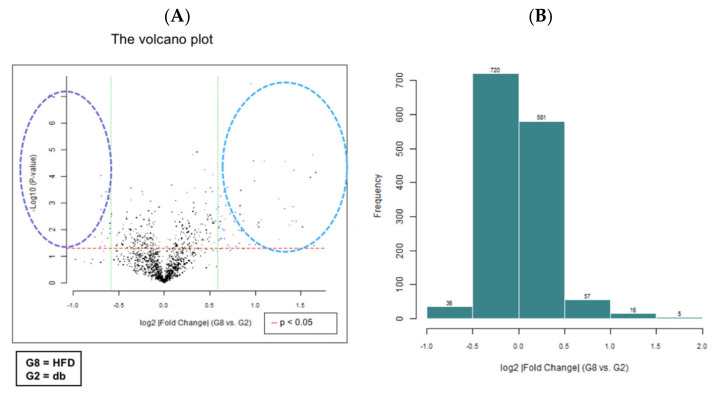
miRNA microarray validation with qRT-PCR analysis in liver tissue samples from grouped HFD-fed db/db mice and standard diet-fed db/db mice. The 6-week-old male db/db mice were fed with HFD for 12 weeks. After euthanization, liver tissue samples were obtained for the determination of miRNA expression. miRNAs were selected from the grouped miRNA microarray dataset and examined through qRT-PCR. The miRNA were sorted on the basis of log2 fold changes in miRNA expression and were represented as horizontal bars (red line in figure). (**A**) The volcano plot of liver miRNA expression in HFD (G8) versus db (G2). Standard selection criteria to identify differentially expressed genes were established at log2 |fold change| ≥ 0.585 and a *p*-value of < 0.05 (blue dots in figure). (**B**) Histogram of log2 |fold change| (HFD versus db). Error bars represent the standard deviation of the mean ± SD. db, db/db mice fed with standard diet; HFD, db/db mice fed with HFD.

**Figure 7:**
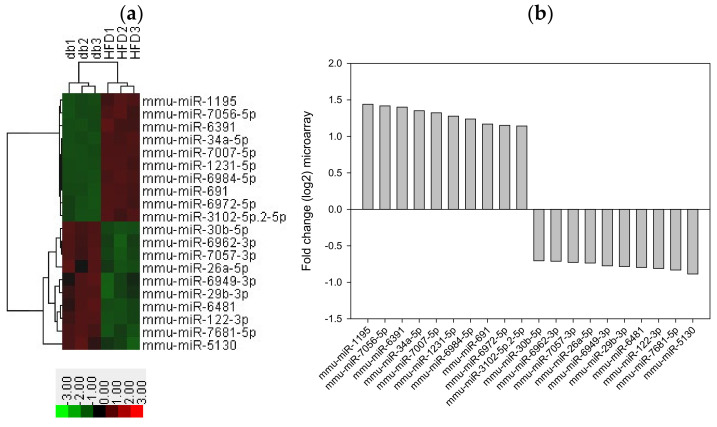
Top 10 upregulated and downregulated miRNA expression in the liver tissue from HFD-fed db/db mice. (**a**) A subset of differential genes was selected for clustering analysis. (**b**) Representation of top 10 upregulated and downregulated genes in red and green colors. miRNAs were detected both by microarray analysis between HFD-fed db/db mice and standard diet-fed db/db mice. The corresponding log2 fold changes in miRNA abundance for each miRNA expression level as determined by microarray analysis are shown.

We performed miRNA microarray validation (The Mouse & Rat miRNA OneArray^®^, Phalanx Biotech Group, Hsinchu, Taiwan) and qRT-PCR analysis to compare the expression of miRNAs in the HFD-fed db/db mice and standard diet–fed db/db mice [23]. Liver tissue samples were obtained for determining miRNA expression. miRNAs were selected from the grouped miRNA microarray dataset, and qRT-PCR was used to compare the miRNA expression between the HFD-fed db/db mice and standard diet–fed db/db mice. As shown in Figure 6A, miRNAs were sorted on the basis of log2 fold changes in their expression and were represented as horizontal bars (red line). Standard selection criteria to identify differentially expressed genes were established as log2 |fold change| ≥ 0.585 and a *p*-value of < 0.05 (blue dots) [24,25] between the HFD (G8) and db (G2) groups. Histograms of log2 |fold change| (HFD vs. db) and gene numbers are presented in Figure 6B. A heatmap showing the expression of the top 10 upregulated and downregulated genes in the liver of the HFD-fed db/db mice is shown in Figure 7.

### 3.5. Search for Predicted miRNA Target Genes through Web-Based Bioinformatic Analysis

We used online database searching tools for miRNA sequence–based prediction, including miRanda (http://www.microrna.org) assessed on 22 January 2018, RNA22 (https://cm.jefferson.edu/rna22) assessed on 20 January 2018, and TargetScan (http://www.targetscan.org) assessed on 20 January 2018, for sorting miRNA candidates and potential miRNA target genes for diabetic steatohepatitis [26,27].

**Figure 8:**
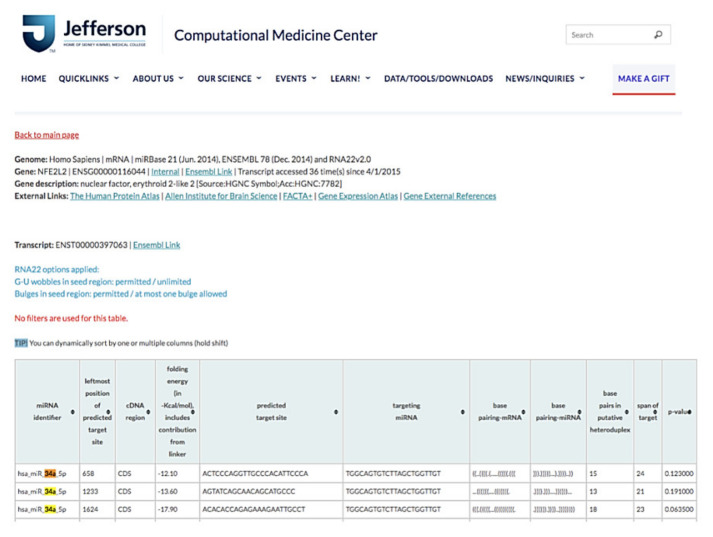
miR-34a-5p interacted with NFE2L2 mRNA to promote its posttranscriptional degradation. Based on RNA22 prediction, the CDS of human NFE2L2 contains one potential seed site for hsa-miR-34a-5p targeting.

The results of RNA22 database analysis indicated that the coding sequence of human *NFE2L2* contains one potential seed site for hsa-miR-34a-5p targeting, suggesting that *NFE2L2* can be a target gene of miR-34a-5p (Figure 8). Because miR-34a-5p was one of the top 10 upregulated genes in our miRNA microarray validation and because a previous study elucidated the protective role of NRF2 for NAFLD [28], we determined whether miR-34a-5p mediated cellular lipid accumulation by targeting NFE2L2 in high-glucose (HG) + O/P-treated HepG2 cells.

### 3.6. miR-34a-5p Inhibitor Ameliorated Lipid Accumulation in HepG2 Cells

**Figure 9:**
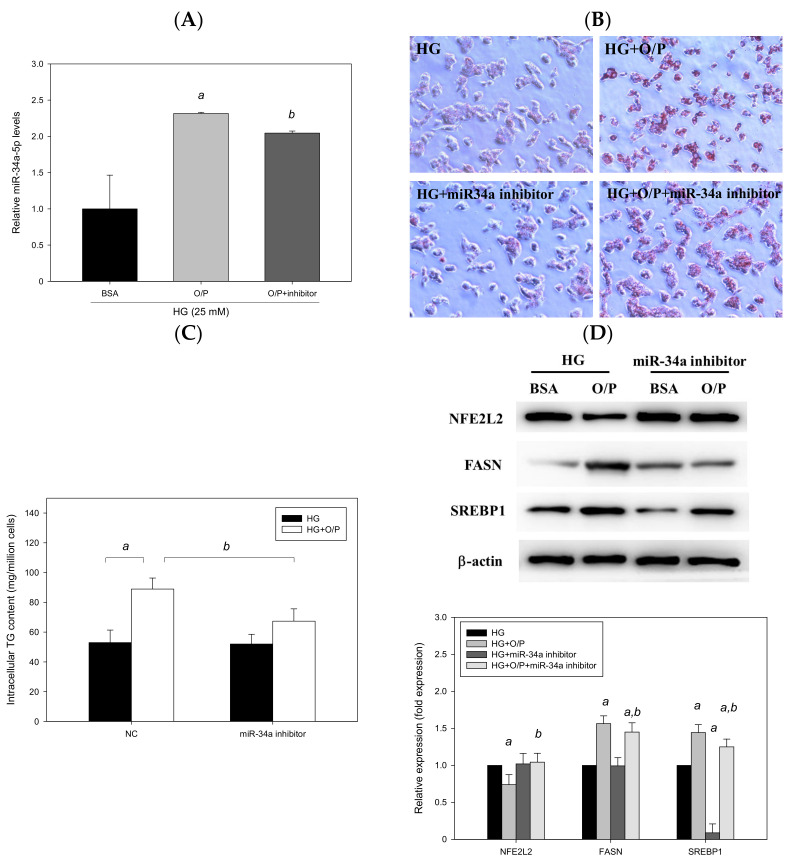
Suppression of miR-34a-5p expression ameliorated oleic acid/palmitic acid mixture-induced lipid accumulation in HG-treated HepG2 cells. A–D, HepG2 cells were transfected with an miR-34a-5p inhibitor or NC for 48 h in the presence of oleic acid/palmitic acid. (**A**) Real-time PCR analysis of miR-34a-5p expression. (**B**) Oil Red O staining. (**C**) Intracellular triglyceride level. (**D**) Western blotting analysis of NFE2L2, FASN and SREBP1. a, *p* < 0.05 compared with the HG control group. b, *p* < 0.05 compared with the HG + O/P group.

We employed HepG2 cells as a model and treated HepG2 cells with HG and a mixture of O/P (2:1, M/M). HG + O/P–induced lipid accumulation was observed through Oil Red O staining (Figure 9B), and the intracellular triglyceride level increased by 75% (*p* < 0.05, Figure 9C), accompanied by the upregulated expression of FASN and SREBP1 observed in Western blotting (Figure 9D). Accordingly, the HG + O/P HepG2 cell model was established to mimic concurrent T2DM and NAFLD pathological conditions. In this model, miR-34a-5p was overexpressed by 132% through treatment with the O/P mixture in HG-treated HepG2 cells (*p* < 0.05, Figure 9A). To determine the importance of miR-34a-5p, HepG2 cells were transfected with an miR-34a-5p inhibitor for 48 h. As shown in Figure 9A, HG + O/P–induced miR-34a-5p overexpression was significantly suppressed by the miR-34a-5p inhibitor. Decreased lipid accumulation was observed through Oil Red O staining after the addition of the miR-34a-5p inhibitor (Figure 9B). Silencing of miR-34a-5p in HepG2 cells ameliorated the HG + O/P–induced elevation of cellular triglycerides by 24% (*p* < 0.05, Figure 9C). Regarding hepatic lipid metabolism–related proteins, the miR-34a-5p inhibitor downregulated the expression of FASN and SREBP1 by 7.24% and 13%, respectively, in HG + O/P HepG2 cells. Moreover, the expression of NFE2L2 was upregulated by the miR-34a-5p inhibitor (*p* < 0.05, Figure 9D). Taken together, these findings indicated that in cells treated with the HG and O/P mixture, miR-34a-5p inhibition improved lipid accumulation, reduced intracellular triglycerides, downregulated hepatic lipid metabolism–related protein expression, and upregulated the expression of NFE2L2.

### 3.7. miR-34a-5p Mimics Affected Lipid Accumulation in HepG2 Cells

**Figure 10:**
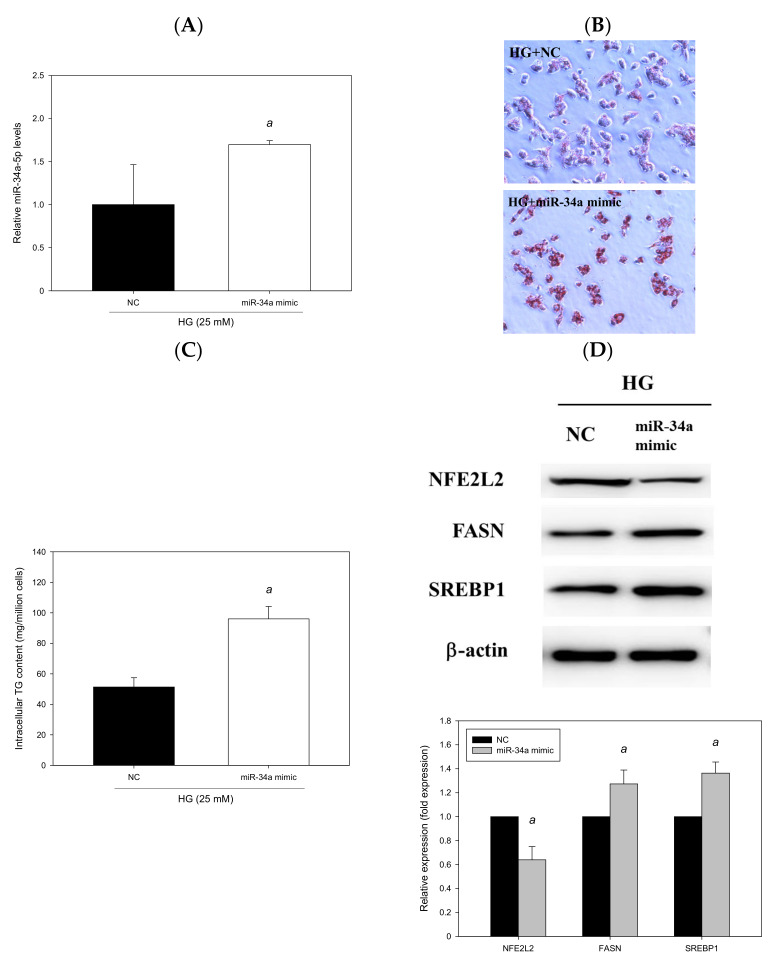
miR-34a-5p induced cellular lipid accumulation in HepG2 cells. HepG2 cells were transfected with an miR-34a-5p mimic or NC for 48 h. (**A**) Real-time PCR analysis of miR-34a-5p expression. (**B**) Oil Red O staining. (**C**) Intracellular triglyceride level. (**D**) Western blotting analysis of NFE2L2, FASN and SREBP1. a, *p* < 0.05 compared with the NC control group.

To explore whether miR-34a-5p can induce cellular lipid accumulation, HG-treated HepG2 cells were transfected with an miR-34a-5p mimic for 48 h. The results revealed that miR-34a-5p was overexpressed by 131% (*p* < 0.05, Figure 10A). The miR-34a-5p mimic significantly induced hepatic lipid accumulation, as observed through Oil Red O staining (Figure 10B), and increased intracellular triglyceride levels (*p* < 0.05, Figure 10C). Moreover, the findings of Western blotting indicated that the expression levels of FASN and SREBP1 were enhanced by 27% and 36%, respectively, in HG-treated HepG2 cells transfected with an miR-34a-5p mimic; and the miR-34a-5p mimic downregulated the expression of NFE2L2 (*p* < 0.05, Figure 10D). Taken together, the findings indicated that the enhancement of miR-34a-5p expression increased lipid accumulation and intracellular triglyceride levels, upregulated hepatic lipid metabolism–related protein expression, and downregulated the expression of NFE2L2. The effects of the miR-34a-5p mimic were compatible with those observed after treatment with O/P in HG-treated HepG2 cells and corresponded to the results of our in vivo experiment conducted in the HFD-fed db/db mice (diabetic steatohepatitis model).

### 3.8. miR-34a-5p Mediated Cellular Lipid Accumulation by Directly Targeting NFE2L2 in HepG2 Cells

**Figure 11:**
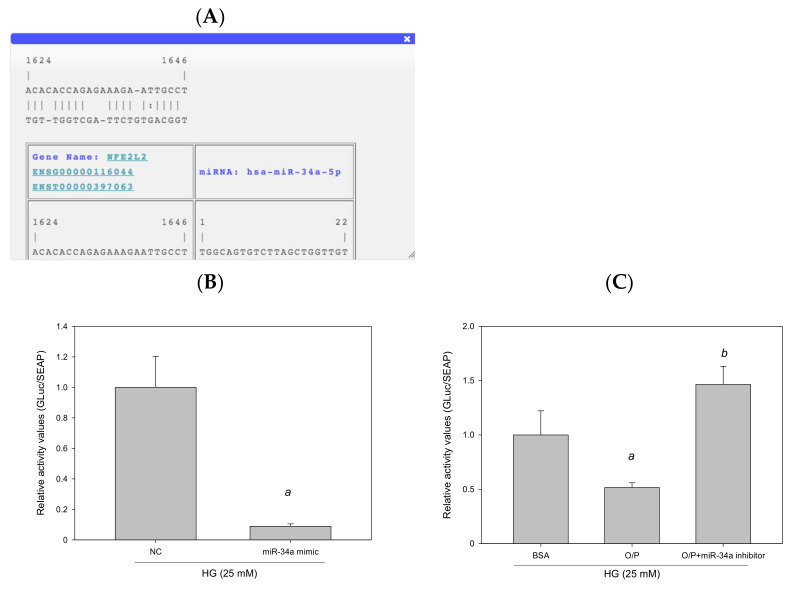
miR-34a-5p mediated cellular lipid accumulation by directly targeting NFE2L2 in HepG2 cells. (**A**) A potential interacting site in the human NFE2L2 was predicted by RNA22. (**B**,**C**) Luciferase reporter plasmids (1 μg) containing the wild type of human NFE2L2 were cotransfected with 40 nM control mimic (NC), 40 nM hsa-miR-34a-5p, 20 pM control inhibitor (O/P), and 20 pM miR-34a-5p inhibitor into HepG2 cells plated in 12-well plates. After 24 h, luciferase activity was measured using the Secrete-Pair *Gaussia* Luciferase Assay Kit. a, *p* < 0.05 compared with the normal control group (NC or BSA group). b, *p* < 0.05 compared with the HG + O/P group.

The results of RNA22 database analysis revealed that the promoter of human *NFE2L2* contains one potential seed site for hsa-miR-34a-5p targeting (Figure 11A). To identify whether NFE2L2 is a true target of miR-34a-5p to determine the interaction between NFE2L2 mRNA and miR-34a-5p, we generated a *Gaussia* luciferase reporter plasmid containing the 3′-UTR of human *NFE2L2* with the miR-34a-5p binding site. The results of the reporter assay performed using HepG2 cells demonstrated that the overexpression of miR-34a-5p induced by the miR-34a-5p mimic reduced luciferase activity by 91% (*p* < 0.05, Figure 11B). Similarly, cotransfection with a control inhibitor (O/P) induced miR-34a-5p expression, thus reducing luciferase activity by 48%. Furthermore, luciferase activity was enhanced by 184% (*p* < 0.05, Figure 11C) after miR-34a-5p expression by an miR-34a-5p inhibitor in HepG2 cells. Taken together, these findings suggest that hsa-miR-34a-5p mediated cellular lipid accumulation by directly targeting NFE2L2 in HepG2 cells.

### 3.9. GA Improved HG + O/P-Induced Lipid Accumulation by Downregulating miR-34a-5p in HepG2 Cells

**Figure 12:**
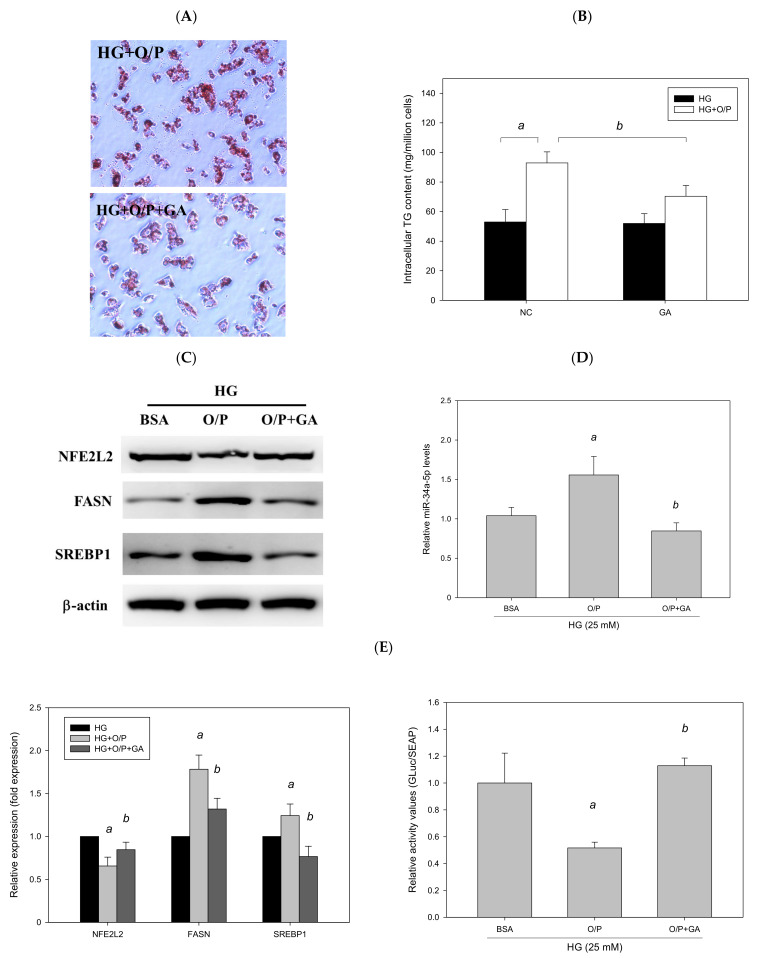

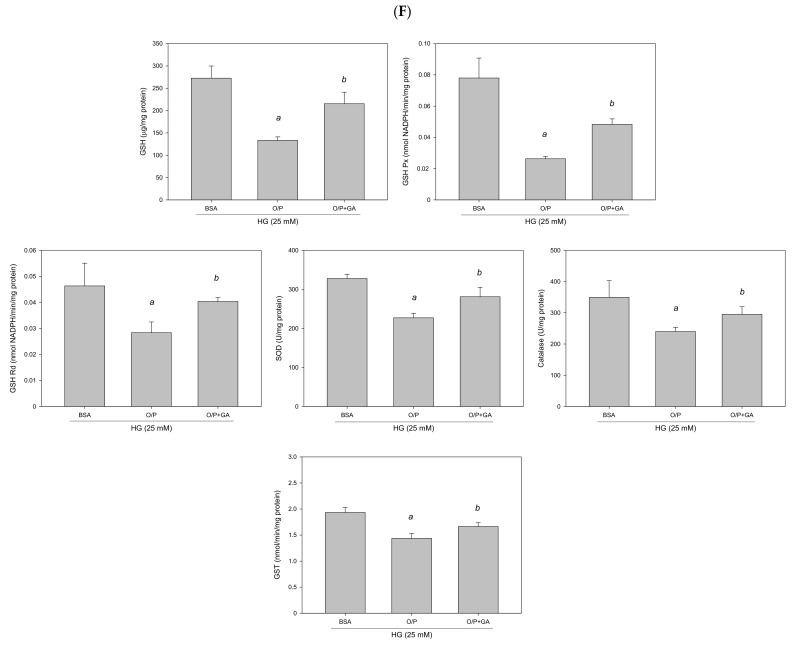
GA-induced reduction in hepatic lipids and lipogenic protein expression is associated with suppression of miR-34a-5p expression in HepG2 cells. HepG2 cells were treated with 10 μM GA for 24 h in the presence of oleic acid/palmitic acid. (**A**) Oil Red O staining. (**B**) Intracellular triglyceride level. (**C**) Western blotting analysis of NFE2L2, FASN, and SREBP1. (**D**) Real-time PCR analysis of miR-34a-5p expression. (**E**) Luciferase activity. (**F**) Antioxidant enzyme activities. a, *p* < 0.05 compared with the normal control group (NC or BSA group). b, *p* < 0.05 compared with the HG + O/P group.

To examine whether GA ameliorated HG + O/P–induced lipid accumulation in HepG2 cells, HG-treated HepG2 cells were treated with GA in the presence of O/P. GA treatment decreased hepatic lipid accumulation, as observed through Oil Red O staining (Figure 12A), and reduced the HG + O/P–induced intracellular triglyceride level by 46% (*p* < 0.05, Figure 12B). In addition, GA modulated hepatic lipid metabolism–related proteins by reducing the expression levels of FASN and SREBP1 (*p* < 0.05, Figure 12C). Taken together, the results showed that GA could alleviate HG + O/P-induced lipid accumulation in HepG2 cells through the attenuation of some lipogenic enzyme levels.

To determine whether GA can improve lipid accumulation by modulating miR-34a-5p in HepG2 cells, we measured miR-34a-5p expression levels. The results revealed that GA significantly reduced miR-34a-5p expression enhanced by the HG and O/P mixture (*p* < 0.05, Figure 12D). The levels of hepatic antioxidant enzymes including GSH, GSH Px, GSH Rd, SOD, catalase, and GST were significantly enhanced by GA treatment (*p* < 0.05, Figure 12F). Moreover, GA increased the expression of NFE2L2 (*p* < 0.05, Figure 12C) and *NFE2L2* luciferase reporter gene analysis demonstrated that GA significantly increased luciferase activity by 184% (*p* < 0.05, Figure 12E), which was reduced by the HG and O/P mixture. These results suggest that GA effectively enhanced antioxidant enzymatic activities, increased the expression of NFE2L2, and improved HG + O/P-induced lipid accumulation by downregulating miR-34a-5p expression and directly targeting NFE2L2 in HepG2 cells.

## 4. Discussion

In this study, we identified a crucial role of miR-34a-5p in oxidative responses and metabolism dysregulation associated with hepatic lipid accumulation in HFD-fed diabetic mice. Furthermore, we established NFE2L2 as a direct target of miR-34a-5p and demonstrated that GA inhibited lipid accumulation and ameliorated steatosis along with reducing miR-34a-5p expression in HepG2 cells. This finding suggests that miR-34a-5p-NFE2L2 is a novel pathway involved in diabetic fatty liver disease. These insights may facilitate the development of innovative therapies for diabetic steatohepatitis that target upstream molecules at the miRNA level.

In this study, HFD substantially affected hepatic lipid accumulation. The HFD-fed db/db mice exhibited impaired liver function and increased plasma cholesterol and triglyceride levels. In addition, the histopathological examination revealed increased accumulation of polymorphonuclear leukocytes and adipocytes leading to hepatic steatosis. As a member of the SREBP family and a crucial regulator of fatty acid metabolism in the liver, SREBP-1c predominantly activates fatty acid and triglyceride biosynthesis by upregulating the transcription of the lipogenic gene *FASN* [29], whereas SREBP2 primarily enhances cholesterol biosynthesis. In our study, HFD increased the TBARS level, reduced antioxidative effects, and increased FASN and SREBP1 expression. In brief, in the db/db mice, HFD contributed to hepatic lipogenesis involved in different pathways including driving oxidative stress, initiating lipid peroxidation, and activating lipogenic enzymes. Accordingly, we established a mouse model of diabetic steatohepatitis. These results are consistent with those of a previous study [7] reporting an increase in the hepatic triglyceride level and FASN and SREBP1 expression, as observed in Western blotting in db/db mice and HFD-fed mice in this study.

Many types of natural products have been used for treating metabolic diseases, and several bioactive compounds such as plant phenolics are beneficial for health. GA, classified as a phenolic acid, is naturally abundant in fruits, vegetables, and herbal medicines. GA possesses antioxidative, anticancer, anti-inflammatory, and antimicrobial properties [30]. GA could reduce oxidative stress and increase GSH, GSH Px, GSH Rd, and GST levels in the hepatic tissues of HFD-induced obese rats. In addition, GA ameliorated hyperlipidemia by reducing hepatic triglyceride and cholesterol levels, thus suppressing hepatic steatosis [31]. A recent study reported that GA alleviated impaired lipid homeostasis and decelerated NAFLD progression in a combined HFD and streptozotocin-induced mouse model [32]. In our model of diabetic steatohepatitis, GA treatment significantly improved impaired liver function and hyperlipidemia, reduced lipid peroxidation and excessive lipid storage, increased antioxidant enzymatic activity, and inhibited lipogenic enzymes in the HFD-fed db/db mice.

Insulin resistance enhances the lipolysis of triglycerides and inhibits the esterification of free fatty acids within adipocytes, thus leading to the increased production of free fatty acids, which are taken up by the liver [33]. Obesity, characterized by an uncontrolled inflammatory response, decreased antioxidative capacity, compromised insulin sensitivity, and dysfunctional angiogenesis, is associated with metabolic diseases including insulin resistance, T2DM, and cardiovascular diseases. The favorable effects of GA on oxidative stress in obesity have been reported [34]. Glucotoxicity refers to the detrimental effects of chronic exposure to hyperglycemia that can lead to cellular dysfunction and irreversible pancreatic β-cell damage characterized by defective insulin gene expression. Both loss of insulin gene expression and excessive apoptosis of β-cells with resultant glucotoxicity in T2DM can be largely attributed to chronic oxidative stress [35]. In our study, GA treatment did not significantly reduce the plasma glucose level in the HFD-fed diabetic mice, as determined by the HbA1c test that measures the average amount of glucose attached to hemoglobin over the past 3 months based on the lifespan of red blood cells [36]. Although GA caused a minimal decline in the glucose level, it suppressed HFD-induced glucotoxicity and enhanced antioxidant enzymatic activities in vivo and in vitro. GA exerts therapeutic effects on diabetic steatosis possibly by reducing lipid peroxidation, inhibiting fatty acid synthesis, enhancing antioxidative activity, attenuating glucotoxicity, and improving hyperinsulinemia [18]. Taken together, the findings indicated that GA could reverse and ameliorate HFD-induced hepatic lipogenesis through the pathways involved in lipid peroxidation, oxidative stress, and the regulation of fatty acid synthesis and lipid homeostasis. Additional studies should explore molecular or epigenetic mechanisms underlying the therapeutic effects of GA on diabetes-related dysfunction; especially, its role in adipogenesis, insulin signaling, and oxidative damage within adipocytes or hepatocytes should be examined.

A study reported GA could ameliorate oxidative stress and inflammation in diabetic rats by modulating miRNA expression [37]. Therefore, we explored whether GA exerts protective effects through the modification of miRNAs involved in hepatic lipid metabolism. In our in vitro experiment, we observed that the GA-induced reduction in intracellular triglyceride and lipogenic enzyme levels was mediated through the suppression of miR-34a-5p expression in HepG2 cells. Our findings suggest that GA can serve as a therapeutic agent against diabetic fatty liver disease by downregulating miR-34a-5p expression.

miRNAs, which possess the ability to control gene expression, are aberrantly expressed in multifactorial diseases including metabolic disorders. Thus, investigating cell-specific miRNA expression or circulating miRNA features can offer a new perspective and can help develop valuable diagnostic modalities for fatty liver disease [38]. In the HFD-fed mouse model, hepatic miR-291b-3p expression facilitated hepatic lipogenesis by inhibiting AMPKα1 expression; downregulation of hepatic miR-291b-3p expression by an miR-291b-3p inhibitor or metformin prevented hepatic steatosis and lipogenesis [7]. In addition, miR-122, miR-33, miR-21, and miR-34a play vital roles in regulating lipid metabolism in the liver [5]. Inhibition of miR-122 in diet-induced obese mice reduced the plasma cholesterol level and ameliorated liver steatosis, accompanied by reductions in the expression of several lipogenic genes involved in fatty acid and cholesterol metabolism [8]. The intronic miRNAs *hsa-miR-33a* and *hsa-miR-33b* located within *Srebp2* and *Srebp1* genes, respectively, regulate cholesterol metabolism and modulate fatty acid β-oxidation and insulin signaling in the liver, which may be particularly relevant in the setting of metabolic syndrome [39]. Circulating levels of miR-122 and miR-34a correlate with hepatic inflammation and fibrosis, indicating their relevance as suitable miRNA biomarkers of NAFLD and NASH [5].

Bioinformatics is a field of computational science that involves the development of techniques and software tools for processing biological data, especially for large and complex datasets. Microarray-based expression analysis can be performed to initially identify candidate miRNAs that are correlated with biological pathways, such as carcinogenesis; these candidate miRNAs can be eventually developed as a molecular signature for a disease state [40,41]. In this study, we analyzed up to 1415 different miRNAs based on miRNA microarray validation and observed significant changes in the expression of several miRNAs in the HFD-induced db/db mice. Thus, we examined the top 10 upregulated and 10 downregulated miRNAs that responded to HFD compared with the standard diet. miR-1195, miR-7056-5p, miR-6391, and miR-34a-5p were among the top 10 upregulated genes in our miRNA microarray validation. miR-1195 was shown to be positively regulated by the transcription factor NK2 homeobox in lung epithelial cells in mice, but not in human cells [42]. miR-6391 and miR-34a-5p were reported to be involved in stearic acid–related lipotoxicity and β-cell dysfunction in mouse pancreatic cells [43]. RNA22 is a pattern-based method for identifying miRNA binding sites and their corresponding heteroduplexes. This method can find putative miRNA binding sites in the sequences of interest and can help determine the identity of target miRNAs [27]. The specific binding between miR-34a and the luciferase construct verified the potential posttranscriptional modification of miR-34a-5p in NFE2L2.

miR-34a regulates diabetes-related protein cascades in the insulin signaling pathway, glucose metabolism, and cell proliferation through Akt, phosphatase 1 nuclear targeting subunit (PNUTS), and SIRT1 expression [9,44]. Akt, also known as protein kinase B, regulates glucose transporter 4 (GLUT4) traffic, causing an increase in membrane GLUT4 in skeletal cells [45]. In addition, miR-34a expression was induced in the ageing heart, and miR-34a inhibition alleviated age-associated cell death and fibrosis following myocardial infarction; PNUTS was identified as a novel direct target of miR-34a [46]. miR-34a-5p directly suppressed SIRT1 in HG-stimulated human proximal tubule cells in the tubulointerstitial fibrosis of diabetic nephropathy [47]. Because of the abundance of miR-34a in the liver, miR-34a appears to facilitate lipid synthesis and inhibit fatty acid oxidation and is significantly upregulated in the plasma and liver tissues of patients with NAFLD and NASH [48], indicating its potential as a specific biomarker for the diagnosis of these diseases. Inhibition of miR-34a could alleviate steatosis and suppress lipid accumulation in the mouse NAFLD model by specifically targeting hepatic PPARα and SIRT1 [10]. Finally, miR-34a controls lipid and lipoprotein metabolism by directly regulating its target hepatocyte nuclear factor 4, a liver-enriched transcription factor regulating genes involved in metabolism, cell junctions, differentiation, and proliferation that is implicated in lipid and bile acid synthesis, gluconeogenesis, and amino acid metabolism [49].

Given its role in the modulation of metabolic homeostasis in liver diseases, miR-34a-5p is highly enhanced in fatty liver disease and might regulate lipid metabolism. In the present study, miR-34a-5p was remarkably upregulated both in the mouse model of diabetic steatohepatitis and the HG + O/P HepG2 cell model. Moreover, miR-34a-5p aggravates hepatic lipogenesis by suppressing NFE2L2 expression. In other words, overexpression of miR-34a-5p in hepatocytes induced intracellular oxidative stress and fatty acid synthesis, attenuated antioxidative activity, downregulated the expression of NFE2L2, and contributed to excessive intracellular triglyceride accumulation. By contrast, the use of an miR-34a-5p inhibitor to suppress miR-34a-5p expression effectively reduced lipid accumulation in NAFLD and diabetic steatohepatitis. Because of its antioxidative activity, GA reduced oxidative stress, inhibited fatty acid synthesis, increased the expression of NFE2L2, and alleviated steatosis, thus exerting protective effects against dysfunctional lipid metabolism by directly targeting NFE2L2, which could be partially attributable to the downregulation of miR-34a-5p expression in the liver. Therefore, miR-34a-5p tightly modulated the development and progression of lipid accumulation in obesity-related diabetic steatohepatitis, and GA significantly inhibited hepatic lipogenesis by downregulating miR-34a-5p through targeting NFE2L2 in diabetic mice.

The transcription factor NFE2L2 exerts antioxidative effects by regulating the expression of antioxidant enzyme genes such as GSH Px, GST, SOD, heme oxygenase-1, and NAD(P)H:quinine oxidoreductase-1 [50]. Activation of NFE2L2 signaling modulates the pathways beyond detoxification and cytoprotection, with the largest gene cluster related to lipid metabolism [51]. NFE2L2 participates in hepatic fatty acid metabolism as a negative moderator of genes that promote hepatic lipogenesis, thus mediating crosstalk between antioxidant responses and lipid metabolism in NAFLD [28]. In NFE2L2-knockout mice, loss of NFE2L2 altered the expression of fatty acid metabolism genes and deteriorated the development of NAFLD due to increased hepatocellular oxidative stress [52]. By contrast, the enhanced expression of NFE2L2 induced hepatic antioxidative and detoxification effects and decelerated the progression of hepatic steatosis in mice fed a methionine- and choline-deficient diet [53]. Furthermore, the synthetic oleanolic triterpenoid 1-[2-cyano-3,12-dioxooleana-1,9(11)-dien-28-oyl] imidazolide (CDDO-Im) is a potent activator of NFE2L2 signaling. Treatment with CDDO-Im prevented HFD-induced obesity, adipogenesis, and hepatic lipid accumulation in wild-type mice, but not in NFE2L2-deficient mice [54], indicating a potential therapeutic benefit of the NFE2L2-activating molecule. Taken together, these findings suggested that NFE2L2 could improve hepatic lipogenesis by exerting antioxidative effects.

Hydrogen sulfide (H_2_S), known for its anti-inflammatory, antioxidative, and cytoprotective activities, can protect tissues from liver injury. A previous study reported that miR-34a mediated the NFE2L2 signaling pathway, in which H_2_S is involved, in a rat model. miR-34a overexpression inhibited the expression of NFE2L2 and its downstream target expression, whereas miR-34a downregulation significantly increased the expression of NFE2L2 and enhanced the hepatoprotective effect of H_2_S. H_2_S might facilitate the NFE2L2-mediated signaling pathway through the downregulation of miR-34a expression [55]. This finding is consistent with that of the present study; we identified NFE2L2 as a direct target of miR-34a-5p, suggesting that miR-34a-5p increased intracellular oxidative stress by suppressing NFE2L2 in our diabetic steatohepatitis model. Moreover, GA and some polyphenolic nutraceuticals oxidize H_2_S to thiosulfate and polysulfides, which elicit antioxidative and cytoprotective effects. These results provide evidence for polyphenols on metabolism of reactive sulfur species and many health benefits of polyphenolic nutraceuticals are mediated through increased polysulfide production [56,57].

T2DM and cardiovascular diseases are associated with increased reactive oxygen species production [58]. Excessive oxidative stress is a major pathophysiological factor in the development and progression of NAFLD. Aberrant expression of miRNAs contributed to the dysregulation of lipogenesis, and the endogenous antioxidant defense is mainly coordinated by NFE2L2 [16]. Future studies should investigate the relevance of miR-34a-5p targeting NFE2L2 and evaluate the therapeutic feasibility of GA or its derivatives in the setting of T2DM and steatohepatitis.

## 5. Conclusions

Our study results demonstrated that miR-34a-5p plays a pivotal role in the regulation of hepatic lipid metabolism and is upregulated in the liver tissues of mice with diabetic steatohepatitis. Overexpression of miR-34a-5p increased oxidative stress and enhanced lipogenic enzyme levels, which, in turn, contributed to hepatic lipogenesis by modulating the suppression of its target NFE2L2. An miR-34a-5p inhibitor may exert therapeutic effects on diabetic fatty liver disease. GA and an NFE2L2-activating agent could downregulate hepatic miR-34a-5p expression and thus may be beneficial for treating diabetic steatohepatitis. Detailed future studies elucidating molecular mechanisms at the miRNA level should be performed to determine the efficacy of GA or other potential substances in the prevention and treatment of diabetic fatty liver disease.

## Figures and Tables

**Table 1:** The plasma biochemical parameters and liver weight in mice.

	db/m	db	HFD	GA
**AST (U/L)**	139.67 ± 14.29	160.83 ± 21.85	312.00 ± 30.73 ^ab^	212.00 ± 19.06 ^abc^
**ALT (U/L)**	34.17 ± 7.70	87.67 ± 10.17 ^a^	411.50 ± 30.38 ^ab^	209.33 ± 23.82 ^abc^
**Cholesterol (mg/dL)**	102.67 ± 12.16	139.17 ± 11.13	415.17 ± 52.13 ^ab^	367.33 ± 44.08 ^abc^
**TG (mg/dL)**	75.17 ± 12.14	98.00 ± 25.61	259.00 ± 81.84 ^ab^	194.50 ± 22.20 ^abc^
**HbA1c (%)**	4.47 ± 0.71	8.23 ± 1.40 ^a^	8.30 ± 0.88 ^a^	8.10 ± 1.33 ^a^
**Insulin (pg/L)**	1.03 ± 0.09	5.24 ± 0.37 ^a^	7.26 ± 1.83 ^ab^	5.20 ± 0.51 ^ac^
**Liver weight (g)**	1.30 ± 0.20	2.27 ± 0.07 ^a^	4.75 ± 0.26 ^ab^	4.69 ± 0.26 ^ab^

db/m, age-matched heterozygous mice fed with standard diet; db, db/db mice fed with standard diet; HFD, db/db mice fed with HFD; GA, db/db mice fed with HFD and GA. AST, aspartate aminotransferase. ALT, alanine aminotransferase. TG, triglyceride. HbA1c, glycosylated hemoglobin. Each value is expressed as the mean ± SD (n = 3/group). Results were statistically analyzed with ANOVA. ^a^, *p* < 0.05 compared with the db/m control group. ^b^, *p* < 0.05 compared with the db group. ^c^, *p* < 0.05 compared with the HFD group.

## Data Availability

All of the data is contained within the article.
